# Trifunctional synergistically designed 3D-Printed bone scaffold for infectious bone defect therapy: design strategy and clinical translation outlook

**DOI:** 10.3389/fbioe.2026.1877026

**Published:** 2026-07-22

**Authors:** Peijie Zhao, Zewen Qiao

**Affiliations:** 1 Shantou Central Hospital, Shantou, China; 2 Hand and Foot Orthopedics Department, General Hospital of Ningxia Medical University, Yinchuan, China; 3 Ningxia Medical University, Yinchuan, China

**Keywords:** 3D printing/additive manufacturing, antibacterial-osteogenic-angiogenic synergy, antimicrobial bone scaffold, clinical translation, infected bone defect, multifunctional biomaterials

## Abstract

**Objective:**

Infection-induced bone defects are a challenging disease in orthopedic clinical practice. Traditional treatments face core problems such as limited bone sources, high risk of antibiotic systemic application-induced resistance, and difficulties in synchronizing infection control and bone regeneration.

**Method:**

3D printing technology, with its advantages of personalized customization, precise structural regulation, and compatibility and adaptability of multiple materials, has become the core preparation method for repair scaffolds for infection-induced bone defects. Constructing an integrated scaffold with three functions - antibacterial, osteogenic, and vascularization - that is temporally coupled and spatially stratified, and oriented towards clinical translation is a key direction to break through the treatment bottleneck of this disease.

**Results:**

This review differs from existing reviews that only focus solely on antibacterial materials, 3D printing scaffold preparation, or bone regeneration mechanisms. It is the first to systematically construct a three-function collaborative framework of “infection control - angiogenesis - bone regeneration” with temporal coupling and spatial stratification. It deeply analyzes the adaptability of different material systems in the infection microenvironment, the logic of selecting anti-infection strategies, and the design rules of biomimetic structures. It comprehensively summarizes the key bottlenecks in clinical translation, real clinical case evidence, and industrialization paths, and clarifies the time-controlled regulatory mechanism and clinical translation targeting path of the three-function collaboration.

**Conclusion:**

3D printed anti-infection bone scaffolds can achieve synchronous repair of infection clearance, bone regeneration, and angiogenesis. The three-function temporal and spatial collaborative design and integrated research for clinical translation are the core development directions in this field, providing theoretical support and practical guidance for the precise treatment of infection-induced bone defects.

## 1 Introduction

Infectious bone defects are severe orthopedic complications resulting from high-energy trauma, postoperative infection after open fractures, and the progression of chronic osteomyelitis (Manoharan et al., 2024). They have a high clinical incidence, long treatment duration, and high disability rate, and are recognized as a difficult-to-treat disease in the global orthopedic field. The incidence of postoperative infection after internal fixation of open fractures is as high as 10%–30% (Abdelgawad et al., 2022). The aging population and high-energy trauma have further driven the incidence of chronic osteomyelitis to increase year by year. Patients often face the risks of repeated debridement, long-term systemic use of antibiotics, non-union of bones, and even amputation, imposing a heavy burden on the medical system and patients’ families (Scholten et al., 2025).

The core clinical contradiction of infectious bone defects lies in the mutual restraint between infection control and bone regeneration: bacterial biofilms can directly inhibit osteoblast activity and disrupt the bone regeneration microenvironment, while long-term systemic use of antibiotics is prone to inducing bacterial resistance, causing liver and kidney function damage and intestinal flora disorder, and failing to balance the dual needs of anti-infection and bone repair (Hemmati et al., 2021). The current mainstream clinical Masquelet technique requires two-stage surgeries to complete, with a treatment duration of several months, and has poor adaptability to large-area and irregular bone defects, making it difficult to achieve simultaneous infection clearance, bone regeneration, and vascularization (Alford et al., 2021; Mathieu et al., 2022). Therefore, developing new bone repair scaffolds that can function stably in the infectious microenvironment and simultaneously achieve multiple repair functions has become a key issue urgently needed to be solved in clinical practice.

3D printing technology, with its advantages such as personalized precise matching, controllable pore structure, compatibility of multi-material composite, and standardized production capabilities, perfectly meets the individualized repair needs of infectious bone defects, making it the preferred technology for preparing multifunctional anti-infective bone scaffolds (Pose-Boirazian et al., 2022). Compared with traditional scaffold preparation methods, 3D printing can precisely replicate the defect morphology based on the patient’s CT/MRI data, simulate the natural bone cancellous microstructure through structural design, and simultaneously load antibacterial, osteogenic, and vascularization active components, providing technical possibilities for integrated repair (Wu et al., 2023).

The repair of infectious bone defects is a complex event involving multiple biological processes (Yin et al., 2025). A single-function scaffold cannot meet clinical needs: antibacterial is the prerequisite for repair, requiring efficient removal of pathogenic bacteria and destruction of biofilms, and establishing an aseptic regenerative microenvironment; osteogenesis is the core of repair, requiring the induction of stem cell directed differentiation and promoting bone matrix mineralization; vascularization is the guarantee of repair, requiring rapid blood supply to avoid necrosis at the center of the scaffold, and simultaneously improving the local delivery efficiency of antibacterial components. More importantly, the three functions do not exist independently but have a coupling relationship in terms of temporal sequence initiation, spatial partitioned coordination, and biological mutual promotion: early efficient antibacterial can protect osteoblasts and vascular endothelial cells from damage, mid-term differentiation and maturation of osteoblasts can promote vascular remodeling, and late mature vascular network can enhance local drug delivery and metabolic waste clearance, further strengthening the anti-infective effect (Zhang et al., 2025; Qu et al., 2025). This spatiotemporal coupled three-function synergy is the core mechanism for the successful repair of infectious bone defects.

At present, there have been numerous reviews published both domestically and internationally on infectious bone defects, 3D printed bone scaffolds, and antibacterial bone repair materials (Jiang and Yu, 2025). However, there are obvious limitations: most of these reviews only focus on a single material, a single antibacterial strategy, or a single bone regeneration function, lacking a systematic analysis of the spatial-temporal coordinated mechanism of antibacterial, osteogenic, and angiogenic functions; some reviews focus on 3D printing technology but do not conduct targeted analyses in response to the specific needs of the infectious microenvironment; most reviews discuss clinical translation at the theoretical level, lacking in-depth discussions on real clinical cases, transformation bottlenecks, and regulatory pathways.

The unique and innovative value of this review lies in three aspects: First, it systematically proposes and analyzes the spatio-temporal coupling and synergistic mechanism of antibacterial, osteogenic, and angiogenic functions, breaking through the limitation of merely parallel listing of functions in previous reviews, and clarifying the temporal expression and spatial hierarchical design logic (Pan et al., 2025); Second, it fully anchors the special needs of the infectious microenvironment, and conducts targeted analyses of materials, strategies, and structures for characteristics such as acidic infectious microenvironment, bacterial biofilms, and inflammatory infiltration (Wu et al., 2022); Third, it takes clinical translation as the ultimate goal, significantly expanding clinical case evidence, real-world application data, industrialization bottlenecks, and solutions, and constructing a complete chain of “basic research - mechanism analysis - clinical translation”. Based on this, this article systematically reviews the material system, antibacterial strategies, printing technology, biomimetic structure, and evaluation system of 3D printed anti-infective bone scaffolds, deeply analyzes the three-function synergistic mechanism and key bottlenecks in clinical translation, looks forward to future development directions, and provides comprehensive, cutting-edge, and practical theoretical references for precise research and clinical application in this field (Wu et al., 2024).

## 2 The material system and anti-infection strategy of 3D-printed anti-infection bone scaffolds

An ideal material for repairing infectious bone defects should maintain good printability, biocompatibility, mechanical stability, degradation compatibility, and be capable of achieving the synergistic activation of antibacterial, osteogenic, and angiogenic functions in a complex infection microenvironment characterized by acidity, high inflammation, and bacterial colonization. This section focuses on the characteristics of the infection microenvironment and conducts in-depth adaptability analysis of the four major types of materials under the infection scenario, clarifying their advantages, disadvantages, and synergistic mechanism, and systematically comparing the applicable scenarios of anti-infection strategies.

### 2.1 Classification, adaptability, and synergistic mechanism of scaffold materials in the infection microenvironment

#### 2.1.1 Synthetic polymer materials

Representative materials: Polylactic acid (PLA), Poly(lactic-co-glycolic acid) (PLGA), Poly(glycolide) (PCL), Polyetheretherketone (PEEK) (Yang et al., 2025). Analysis of infection microenvironment adaptability: These materials have excellent printing properties and degradable materials (PCL, PLGA) can slowly degrade in the infection microenvironment, avoiding the need for secondary surgery for removal; however, a single material has obvious defects in the infection environment: no inherent antibacterial activity, easily adhered by bacteria to form biofilms; lack of osteogenic activity, weak bone integration ability; acidic degradation products may aggravate local inflammatory response. Strategies for infection repair modification: Compositing with HA/β-TCP to enhance osteogenic activity and neutralize acidic products; loading antibiotics and antimicrobial peptides to achieve anti-infection; grafting VEGF and DFO to improve local blood supply. Among them, PCL/HA and PLGA/β-TCP have good stability and high biocompatibility in the infection microenvironment, and are the preferred polymer composite materials for non-bearing and lightly bearing infection-related bone defects (Aubry et al., 2022). Synergistic mechanism: Polymers provide mechanical support and structural formation, ceramic components enhance osteogenesis and neutralize acidic degradation products, antibacterial components remove local bacteria, and the three work together to achieve infection control and bone repair (Mishchenko et al., 2023). The trade-off between comprehensive properties of polymer composites varies significantly across clinical sites. For non-weight-bearing and maxillofacial infectious bone defects, high porosity (60%–85%) is prioritized to guarantee cell infiltration and nutrient exchange; however, increased porosity inevitably reduces mechanical strength, so PCL/HA and PLGA/β-TCP composites are designed with moderate porosity (60%–70%) to balance structural stability and biological performance. Their slow degradation rate matches the long-term bone regeneration cycle of maxillofacial bones, while acidic degradation products can be neutralized by bioceramic phases to avoid aggravating local infection and inflammation. For light-weight-bearing extremities defects, the porosity is controlled at 60%–70% and pore size at 300–500 μm to elevate mechanical strength, and antibacterial components are loaded via surface modification to avoid compromising bulk mechanical properties. Single polymers suffer from an inherent trade-off: improving antibacterial activity by high-dose drug loading will reduce cell biocompatibility, while excessively accelerating degradation will lead to premature structural collapse before bone repair.

#### 2.1.2 Bioceramic materials

Representative materials: Hydroxyapatite (HA), β-tricalcium phosphate (β-TCP), Bioactive glass. Analysis of infection microenvironment adaptability: The chemical composition is highly similar to natural bone, with excellent osteogenic activity and biocompatibility, and is not easily adhered by bacteria in the infection microenvironment, inhibiting the early formation of biofilms; through doping with ions such as Ag^+^, Cu^2+^, Zn^2+^, broad-spectrum antibacterial ability can be obtained, and Zn^2+^, Mg^2+^ have vascularization-promoting functions (Park et al., 2023). The core defect is high brittleness and low mechanical strength, which makes it prone to fracture in bearing areas and cannot be used alone for weight-bearing bone defect repair. Applicable scenarios for infection repair: Non-bearing and lightly bearing infection-related bone defects, can be used as the osteogenic and antibacterial functions of the composite system. Synergistic mechanism: Ceramic matrix provides an osteogenic microenvironment, doping with metal ions to exert antibacterial and vascularization-promoting effects, and achieving “antibacterial - osteogenesis - vascularization” synchronous activation in the infection microenvironment (Okada et al., 2001). Bioceramics exhibit the most prominent mechanical-biological trade-off: high osteogenic activity and broad-spectrum antibacterial performance via ion doping are accompanied by high brittleness. This material is completely inapplicable to weight-bearing femoral and tibial defects, where cyclic mechanical load easily causes scaffold fracture. It is exclusively used for non-weight-bearing maxillofacial, hand and foot defects. Increasing ion doping concentration enhances antibacterial efficiency but triggers cytotoxicity, forming a typical “antibacterial activity-biocompatibility” trade-off; adjusting porosity to over 80% improves cell infiltration but further weakens mechanical properties, which is only acceptable for superficial bone defects.

#### 2.1.3 Metallic materials

Representative Materials: Titanium alloys, magnesium alloys, zinc alloys. Analysis of Adaptability to the Infection Microenvironment: Titanium alloys have excellent mechanical strength and are suitable for bearing bone defects with bone loss, but they do not have inherent antibacterial activity. Their surfaces are prone to form bacterial biofilms and require surface modification. Biodegradable magnesium/zinc alloys exert antibacterial effects in the infection microenvironment through ion release, and their mechanical strength matches that of natural bone. They also possess osteogenic and angiogenic activities, making them ideal materials for bearing infection-related bone defects. The drawback of magnesium alloys is that the degradation rate is too fast, which leads to excessive hydrogen gas production and local alkalization, disrupting the balance of the infection microenvironment and requiring precise control of the degradation rate through alloying. Applicable Scenarios for Infection Repair: Titanium alloys are used for bearing infection-related bone defects (requiring antibacterial coatings on the surface); magnesium/zinc alloys are used for bearing infection-related bone defects that require degradation and absorption. Synergistic Mechanism: The metallic matrix provides mechanical support and structural stability, and the degradation ions simultaneously achieve antibacterial, osteogenic, and angiogenic functions, maintaining structural stability and functional synergy in the bearing infection environment (Fan et al., 2024; Li and Yang, 2024). Metallic materials are the only choice for weight-bearing infectious defects of femur and tibia, where mechanical strength is the primary indicator. Titanium alloy has stable mechanical properties and non-degradable characteristics; its trade-off lies in “surface antibacterial modification vs. interface binding”: thick antibacterial coatings improve anti-infection ability but increase the risk of coating shedding under cyclic load. Biodegradable Mg/Zn alloys face the triple trade-off of degradation rate-mechanical stability-ion release: rapid degradation produces excessive hydrogen and alkaline microenvironment, disrupting infection microenvironment and cell activity; slow degradation retains mechanical strength but delays bone remodeling. The release of antibacterial metal ions is coupled with material degradation, so regulating degradation kinetics is the key to synchronizing antibacterial effect, mechanical support and bone regeneration for weight-bearing bone segments.

#### 2.1.4 Composite materials

Analysis of Adaptability to the Infection Microenvironment: Composite materials are the optimal material system for infection-related bone defect repair, capable of integrating the advantages of individual materials and resolving the core contradiction of “mechanics - antibacterial - osteogenesis - angiogenesis” that is difficult to balance in the infection microenvironment. Typical Systems: Polymer/bioceramic/antibiotic (PLGA/β-TCP/AgNPs), metal/bioceramic (Mg-Zn/HA), multi-component composite systems loaded with VEGF/DFO. Synergistic Mechanism: In the infection microenvironment, polymers/magnesium provide mechanical support and structural stability, ceramic components promote osteogenesis, antibacterial components eliminate bacteria, angiogenic factors accelerate blood vessel reconstruction, and the multi-component system overcomes the deficiencies of individual materials, achieving efficient and three-functional synergy (O'Neill et al., 2017). Composite materials are designed to resolve the multi-performance trade-off above. For weight-bearing bone defects, metal/bioceramic composite systems (Mg-Zn/HA) are adopted to retain high mechanical strength while regulating ion release. For maxillofacial and non-weight-bearing defects, polymer/bioceramic/antibacterial agent composites (PLGA/β-TCP/AgNPs) are selected to balance degradability, porosity and bioactivity.

### 2.2 Classification, mechanism and adaptability to infection scenarios of anti-infection strategies

Anti-infection strategies are the core mechanism by which scaffolds exert their effects in the infected microenvironment. Based on the mechanism of action, they are classified into five categories: antibiotic class, inorganic ion class, biological macromolecule class, physical antibacterial class, and intelligent response class. Different strategies exhibit significant differences in the infected microenvironment and require precise selection based on the type of infection, disease course, and location (Table 1).

① The antibiotic class strategy is represented by vancomycin and gentamicin, which rapidly kill bacteria by inhibiting bacterial cell wall synthesis and protein synthesis (Beenken et al., 2021). It is clinically mature and has a significant effect on acute sensitive bacterial infections; however, it is prone to burst release in chronic infection microenvironments, generating cytotoxicity at high concentrations, and inducing drug resistance with long-term use. It is only recommended for acute sensitive bacterial infections (Zhu et al., 2025).
② The inorganic ion class (Ag^+^, Cu^2+^, Zn^2+^, Mg^2+^) exerts broad-spectrum antibacterial effects by destroying bacterial cell membranes and inducing the production of reactive oxygen species (Mu et al., 2024). It does not induce drug resistance and also has osteogenic and vascularization activities, making it suitable for chronic infections, mixed infections, and resistant bacterial infections. However, high-concentration ions have cytotoxicity and the release rate is difficult to control, requiring precise controlled release through material composites (Maiti et al., 2024). In terms of long-term biosafety of inorganic antibacterial ions, Ag^+^, Cu^2+^, Zn^2+^ and Mg^2+^ released during scaffold degradation will enter the local tissue fluid and blood circulation in a sustained manner. Low-concentration ions can be metabolized by the liver and kidneys and excreted through urine and feces without long-term tissue accumulation (Mu et al., 2024). However, excessive residual ions in local bone tissue after complete scaffold degradation may cause chronic cytotoxicity, interfere with osteoblast metabolism, and even affect adjacent soft tissue function. Current studies confirm that doping concentration below 5 mol% can effectively avoid cumulative toxicity during long-term follow-up. For degradable metal and ceramic scaffolds, metal ions will be gradually consumed and metabolized synchronously with scaffold degradation and new bone ingrowth, and no abnormal ion accumulation was observed in 12-month long-term animal experiments (Wu et al., 2026). At present, the long-term biosafety data beyond 2 years is still insufficient, which is one of the key risks restricting large-scale clinical application.
③ The biological macromolecule class (antibacterial peptides, chitosan, lysozyme) has excellent biocompatibility, can destroy bacterial cell membranes and inhibit biofilm formation, has a low risk of drug resistance, is biodegradable, and is suitable for mild infections, pediatric patients, and soft tissue-bone composite defects (Shen et al., 2023). However, the preparation cost is high and the stability is poor, and it is prone to inactivation in the infected inflammatory microenvironment, requiring the use of carriers to increase the half-life.
④ The physical antibacterial class (graphene, black phosphorus, photocatalytic materials) achieves antibacterial effects through photothermal effects, photocatalytic oxidation, and physical puncturing of the cell membrane (Kavitha Sri et al., 2023). It has no drug resistance and can be remotely regulated, and has both antibacterial and osteogenic functions. However, it requires external light stimulation, has limited penetration depth, and has a risk of thermal damage, and is only suitable for superficial bone infections, recurrent infections, and biofilm infections.
⑤ The intelligent response class (pH/enzyme/bacterial trigger release) can respond to the acidic environment of the infected microenvironment and high enzymatic characteristics to achieve targeted and on-demand release, reducing the dosage and toxicity, and reducing drug resistance (Moghaddam et al., 2024); however, the preparation process is complex and costly, and clinical translation is not mature, and it is the future direction for chronic recurrent infections and personalized intelligent treatment. Typical practical examples of stimulus-responsive release mechanisms are as follows: (1) pH-triggered release: Using PLGA microspheres as carriers, the material swells and ruptures under the acidic microenvironment (pH = 5.0–6.0) of bacterial infection, and releases vancomycin and antimicrobial peptides on demand; this system has been verified in MRSA-infected bone defect models (Zhuo et al., 2020). (2) Enzyme-triggered release: Chitosan hydrogel carriers respond to high-concentration matrix metalloproteinases (MMPs) secreted in infected tissues, realizing targeted degradation and drug release. (3) Bacteria-triggered release: Scaffold surface modified with bacterial exoenzyme-responsive peptides, which are specifically cleaved by bacterial secretions to activate antibacterial components, avoiding drug leakage in normal tissues (O'Neill et al., 2017). The above three trigger modes are the most mature intelligent response mechanisms for infectious bone defects at present.

**TABLE 1:** Comparison of different anti-infection strategies for 3D printed bone supports.

Anti-infective strategy	Typical active ingredients	Core mechanism of action	Advantages	Limitations	Applicability in infectious scenarios	Typical research cases	Experimental conditions & results
Antibiotic-based	Vancomycin, Gentamicin	Inhibit bacterial cell wall/protein synthesis	High bactericidal efficiency, mature clinical application	High risk of inducing drug resistance, burst release effect, cytotoxicity at high concentration	Acute sensitive bacterial infection, early infection control	3D printed titanium alloy scaffold loaded with vancomycin	Clinical trial for chronic osteomyelitis; Local sustained drug release, 12-month infection recurrence rate <5%, effective for acute *Staphylococcus aureus* infection
Inorganic ion-based	Ag^+^, Cu^2+^, Zn^2+^, Mg^2+^	Disrupt bacterial cell membrane, induce ROS production, inhibit enzyme activity	Broad-spectrum antibacterial activity, low risk of drug resistance, combined osteogenic/angiogenic activity	Potential cytotoxicity at high concentration, uncontrollable ion release	Chronic infection, mixed infection, drug-resistant bacterial infection	Zn-doped Mg alloy scaffold, AgNPs/β-TCP composite	Animal model of MRSA infection; Ion doping 3–5 mol%, bacteriostatic rate >92%, no obvious cytotoxicity, synchronized osteogenesis and
Biomacromolecule-based	Antimicrobial peptides, Chitosan, Lysozyme	Disrupt bacterial cell membrane integrity, inhibit biofilm formation	Excellent biocompatibility, low drug resistance, biodegradable	High preparation cost, short half-life, poor stability	Mild infection, pediatric patients, soft tissue-bone composite defects	Chitosan/lysozyme composite hydrogel scaffold	*in vitro* mild bacterial infection model; Biofilm inhibition rate >85%, good compatibility with primary osteoblasts, suitable for pediatric superficial bone infection
Physical antibacterial	Graphene, Black phosphorus, Photocatalytic materials	Photothermal heating, photocatalytic ROS production, physical puncture of cell membrane	No drug resistance, remote controllability, combined antibacterial-osteogenic function	Requires external light stimulation, limited penetration depth, risk of tissue thermal damage	Superficial bone infection, recurrent infection, biofilm infection	Black phosphorus modified PCL scaffold	808 nm near-infrared light stimulation; Superficial bone infection model, bacteriostatic rate >90%, no thermal damage to peripheral normal tissues
Smart responsive	pH/enzyme/bacteria-triggered release system	Targeted release in response to infectious microenvironment (acidic, high enzyme level)	Precise on-demand drug delivery, low dosage, reduced toxicity and drug resistance	Complex preparation process, high cost, immature clinical translation	Chronic recurrent infection, personalized intelligent therapy	pH-responsive PLGA/antibiotic microsphere scaffold	Acidic infection microenvironment (pH 5.2); On-demand drug release, drug dosage reduced by 40%, effectively suppress recurrent infection in animal models

This table systematically compares the antibacterial mechanism, merits, limitations, applicable clinical scenarios and representative research evidence of five mainstream anti-infection strategies for 3D-printed bone scaffolds targeting infectious bone defects. All *in vitro* bacteriostatic indexes and animal model outcomes cited in the table are derived from published peer-reviewed studies.

### 2.3 Antibacterial - osteogenic - angiogenic three-function spatial synergistic design mechanism

The three-function synergy is not a simple superposition of functions, but a coupled process that is initiated sequentially in time, acts separately in space, and promotes each other biologically. It is the core scientific issue in the repair of infectious bone defects.

Temporal Synergistic Mechanism: The repair of infectious bone defects follows the physiological rule of “antibacterial first, then angiogenesis, and finally osteogenesis”. The scaffold releases antibacterial components preferentially in the early stage (0–2 weeks), quickly eliminating pathogenic bacteria, destroying biofilms, and creating a sterile microenvironment to prevent damage to osteogenic and vascular endothelial cells; in the middle stage (2–8 weeks), the antibacterial components are maintained at a low dose, and angiogenic factors are released in large quantities, promoting the growth of new blood vessels into the scaffold and providing oxygen and nutrients for cells; in the later stage (8–24 weeks), the vascular network matures, osteogenic signals are activated, stem cells differentiate directionally, and bone matrix mineralization occurs, achieving complete repair of bone defects (Wu et al., 2026).

Spatial Synergistic Mechanism: A gradient pore structure and core-shell structure are used to achieve spatial separation (Kareem et al., 2019). The small pores on the outer layer of the scaffold (200–400 μm) load antibacterial components, preventing bacterial invasion and quickly clearing surface bacteria; the large pores on the inner layer (500–800 μm) load osteogenic and angiogenic factors, promoting cell infiltration, vascular growth, and bone tissue regeneration, forming a spatial synergistic mode of “outer layer antibacterial, inner layer repair”.

Biological Coupling Mechanism: The antibacterial function clears bacteria, inhibits inflammation, protects the activity of osteogenic cells and vascular endothelial cells, laying the foundation for repair; osteogenic cells secrete vascular endothelial growth factor, promoting vascular remodeling and maturation; new blood vessels improve local microcirculation, enhance the delivery efficiency of antibacterial components, and strengthen the anti-infection effect, forming a positive cycle of antibacterial protection for osteogenesis, osteogenesis promoting angiogenesis, and angiogenesis strengthening antibacterial (Wojdasiewicz et al., 2024).

Based on these mechanisms, the material design should follow four major paths: ① Multi-component composite, integrating antibacterial agents, osteogenic ceramics, and angiogenic factors into a single scaffold; ② Selecting inherent three-function materials (such as Mg-Zn alloy); ③ Gradient/core-shell structure for spatial partitioning; ④ Controlled-release technology for temporal expression to maximize the synergistic effect.

## 3 3D printing technology and bionic structure design

### 3.1 Mainstream 3D printing technologies and clinical applicability for infectious bone defects

The 3D printing technology directly determines the mechanical properties, pore connectivity, and functional release efficiency of the scaffold in an infected environment. It needs to be selected based on material characteristics, infection site, and load-bearing requirements (Table 2).

**TABLE 2:** Comparison of mainstream 3D printing technologies for bone scaffolds.

Printing technology	Full English name	Applicable materials	Printing accuracy	Core advantages	Limitations	Clinical applicability in infectious bone defects	Technical maturity	Specific clinical cases & evidence
FDM	Fused Deposition Modeling	PLA, PCL, polymer composites	Low (100–300 μm)	Low cost, fast printing speed, easy operation	Low accuracy, poor surface finish, high risk of bacterial adhesion	Basic research, small animal experiments, non-load-bearing small defect scaffolds	Laboratory & Preclinical	Mainly used for rodent animal experiments; no formal clinical application cases
SLS	Selective Laser Sintering	PEEK, metal/ceramic powders	Medium (50–100 μm)	High mechanical strength, excellent load-bearing stability	High equipment cost, limited material selection	Load-bearing bone infectious defects, chronic osteomyelitis repair	Clinical Application	Used for adult tibial/femoral chronic osteomyelitis repair; PEEK scaffolds with personalized shaping, clinical follow-up shows good structural stability (Goldmann, 2021)
SLA/DLP	Stereolithography/Digital Light Processing	Biocompatible photosensitive resin, ceramic slurry	High (10–50 μm)	Ultra-high precision, complex bionic structure fabrication	Limited material types, post-processing required	Precision bionic scaffolds, small-area irregular infectious bone defects	Clinical Application	Maxillofacial infectious bone defect repair; high-precision matching of irregular defects, osteointegration rate >85% in clinical cases (Qiao et al., 2022)
EBM	Electron Beam Melting	Titanium alloy/Magnesium alloy	Medium-High (20–80 μm)	High relative density, excellent mechanical properties	Extremely high cost, strict operation requirements	Clinical load-bearing metal infectious bone implants	Clinical Small-scale Application	Large segment femoral infectious defects; titanium alloy implants for load-bearing reconstruction, single-center small-sample application (Palmquist et al., 2023)
3D Bioprinting	3D Bioplotting	Hydrogels, cell-laden materials	Medium (30–100 μm)	Mild printing conditions, compatible with active factors/cells	Low mechanical strength	Bioactive scaffolds, non-load-bearing infectious bone tissue engineering repair	Preclinical	Cell-loaded hydrogel scaffolds for hand and foot non-weight-bearing defects; only verified in animal models, no large-scale clinical use

The table summarizes the material adaptability, printing precision, technical advantages and clinical application scope of five common bone scaffold manufacturing techniques. Clinical maturity is divided into three grades: laboratory preclinical testing, small-scale clinical trial and mature clinical application, which is judged based on existing orthopedic implant clinical literature.

Fused Deposition Modeling (FDM) (Iqbal et al., 2024) is applicable to high molecular materials such as PLA and PCL, with low cost and simple operation, suitable for basic research and small animal experimental scaffolds. However, it has low precision and rough surface, and is prone to bacterial adhesion in the infected environment, only used for the preparation of non-bearing and small-sized scaffolds for infectious bone defects.

Selective Laser Sintering (SLS) (Gueche et al., 2021) is applicable to PEEK, metal/ceramic powders, with high mechanical strength, suitable for the preparation of load-bearing scaffolds. It can maintain structural stability in the infected load-bearing environment; however, the equipment cost is high and the material selection is limited, mostly used for clinical load-bearing infectious bone defect repair.

Stereolithography/Digital Light Processing (SLA/DLP) has extremely high precision and can fabricate complex biomimetic structures, suitable for precise small-area bone defects (Atwal and Bhatnagar, 2024); however, the material types are limited and post-processing is required, and it is widely used in the precise repair of infectious bone defects.

Electron Beam Melting (EBM) (Palmquist et al., 2023) has high density and excellent mechanical properties, suitable for titanium alloy and magnesium alloy load-bearing implants, used for the preparation of large clinical load-bearing infectious bone defect scaffolds, but the cost is extremely high and the operation is strict.

3D biological printing uses hydrogels and cell-carrying materials, has mild printing conditions, can load active factors and cells, suitable for the preparation of bioactive scaffolds; but the mechanical strength is low, and it is used for tissue engineering repair of non-load-bearing infectious bone defects.

### 3.2 Biomimetic structural design for tri-functional synergy in the infectious microenvironment

The infectious bone defect site presents a unique pathological microenvironment characterized by persistent inflammation, bacterial colonization, biofilm encapsulation, and microcirculatory disturbance. The topological morphology, pore size distribution, pore connectivity, and interface structure of the scaffold not only determine the mechanical stability and tissue integration efficiency but also directly regulate the release of antibacterial components, bacterial adhesion and colonization, osteoblast behavior, and endothelial cell-mediated angiogenesis. Therefore, the biomimetic structural design targeting antibacterial–osteogenic–angiogenic tri-functional synergy must accommodate both the biomimetic characteristics of natural bone tissue and the tolerance requirements of the infectious microenvironment, so as to achieve a high degree of matching among structure, function, and microenvironment.

Numerous *in vitro* and *in vivo* studies have confirmed that porous scaffolds for bone regeneration should possess pore characteristics similar to those of cancellous bone to ensure mass exchange, cell infiltration, and vascular ingrowth. Zhao et al. (2022) et al. verified through experiments that for the repair of infectious bone defects, the ideal pore parameters should be: pore diameter ranging from 300 to 800 μm, pore volume ranging from 60% to 85%, and pore connectivity greater than 90%.This range not only ensures that the scaffold exhibits mechanical strength matching with host bone to prevent collapse and fracture in load-bearing regions but also provides sufficient space for nutrient transport, metabolic waste diffusion, cell migration, and neovascularization. Meanwhile, it reduces the risk of bacterial adhesion, aggregation, and subsequent biofilm formation on the scaffold surface.

To realize the spatial and temporal synergy of antibacterial, osteogenic, and angiogenic functions, the graded pore structure has become the preferred design in the infectious microenvironment. Zhao et al. (2025) used 200–400 μm-sized holes on the outer layer of the bone scaffoldwhich can increase the local concentration of antibacterial agents, form a physical barrier to block bacterial invasion into the scaffold interior, and improve the surface stability and interfacial bonding capability of the scaffold. The inner layer employs large pores of 500–800 μm, which are more conducive to the infiltration, adhesion, and osteogenic differentiation of bone marrow mesenchymal stem cells. Meanwhile, such large pores provide open pathways for endothelial cell migration, lumen formation, and microvascular network construction, thereby achieving the spatial functional partitioning of antibacterial protection in the outer layer and osteogenic–angiogenic repair in the inner layer.

In terms of topological structure selection, triply periodic minimal surface (TPMS) structures (including Gyroid, Diamond, Primitive, and other configurations) show significant advantages in the repair of infectious bone defects. Compared with traditional orthogonal grids and bead-like pore structures, TPMS structures feature continuous smooth surfaces, no stress concentration, high connectivity, and large specific surface area. They can bear mechanical loads uniformly, reduce the stress shielding effect of the scaffold in the defect area, and improve the loading efficiency and controlled release stability of bioactive factors and antibacterial components. In the infectious microenvironment, this structure can significantly reduce bacterial adhesion, promote effective cell infiltration, and accelerate the inward growth of new blood vessels from the periphery, thus synergistically enhancing the efficiency of antibacterial, osteogenic, and angiogenic repair (Kumar et al., 2025).

Combined with real clinical defect scenarios, the scaffold structure should be further designed with site-specific and functionally adaptive features: for load-bearing sites such as long bones of the lower limbs and spine, a composite structure of *dense surface–porous interior* is recommended, mainly made of high-strength materials such as titanium alloy and PEEK, to ensure mechanical support and structural stability. For non-load-bearing or light-load-bearing sites such as maxillofacial, hand, foot, and ankle regions, degradable polymer–ceramic composites (e.g., PCL/HA, PLGA/β-TCP) are preferred. By regulating the pore structure and degradation rate, the scaffold can dynamically match the rate of bone regeneration, and finally achieve the integrated repair of gradual scaffold degradation and new bone replacement. Combined with the performance trade-off and clinical site characteristics, the matching rules of material system, porous structure and 3D printing technology for different infectious bone defects are summarized as follows.

① Weight-bearing defects (femur, tibia, spine): Preferred materials: titanium alloy, Mg-Zn alloy; Porous structure: dense outer layer + gradient porous inner layer (pore size 300–500 μm), TPMS structure to reduce stress concentration; 3D printing technology: SLS, EBM, to ensure overall mechanical strength and structural integrity under cyclic load.
② Non-weight-bearing/light-weight-bearing defects (hand, ankle): Preferred materials: PCL/HA, PLGA/β-TCP composites; Porous structure: uniform large pores (500–800 μm), connectivity >90%; 3D printing technology: FDM, 3D bioprinting, taking degradability and cell infiltration as the core.
③ Maxillofacial infectious bone defects: Preferred materials: bioactive glass, HA/β-TCP ceramics; Porous structure: outer small pores (200–400 μm) + inner large pores (500–800 μm) core-shell structure; 3D printing technology: SLA/DLP, to realize high-precision personalized shaping for complex anatomical morphology.

## 4 *In vitro* and *in vivo* evaluation system

### 4.1 *In vitro* evaluation of tri-functional synergy

The *in vitro* assessment of 3D-printed anti-infective bone scaffolds should be performed under a simulated infectious microenvironment to rigorously validate the coordination and sustainability of antibacterial, osteogenic, and angiogenic functions (Chen et al., 2024). Antibacterial tests should include the most clinically relevant pathogens, such as *Staphylococcus aureus*, *Pseudomonas aeruginosa*, and methicillin-resistant *S. aureus* (MRSA), in order to evaluate broad-spectrum antibacterial activity and biofilm inhibition/eradication capability. For osteogenic evaluation, bone marrow-derived mesenchymal stem cells (BMSCs) are commonly utilized to assess cytocompatibility, cell adhesion, proliferation, alkaline phosphatase activity, matrix mineralization, and osteogenic gene expression under inflammatory and infectious conditions. Angiogenic performance is typically evaluated using human umbilical vein endothelial cells (HUVECs) by detecting cell migration, tube-like structure formation, and angiogenic gene expression. A critical confirmatory principle is that antibacterial components must not suppress the viability or biological functions of osteogenic and vascular cells; meanwhile, angiogenic factors can improve local microcirculation and further promote the delivery and efficacy of antibacterial agents (Gao et al., 2024).

### 4.2 *In vivo* preclinical evaluation and clinical translation evidence

Based on the research maturity and application scope, the research progress of 3D printed anti-infective bone scaffolds is divided into four categories: experimental concept, preclinical evidence, small-scale clinical application, and approved commercial clinical products.

① Experimental concept (*in vitro* basic research):This category refers to novel material formulas, innovative antibacterial strategies and bionic structural designs only verified by *in vitro* cell and antibacterial tests, including pH-responsive intelligent release scaffolds, photothermal antibacterial TPMS structural scaffolds, and 4D printed shape-memory scaffolds. These technologies remain in the laboratory conceptual stage, without animal model verification (Wang et al., 2020).
② Preclinical evidence (animal model research):Technologies that have completed standardized verification in infectious bone defect animal models (New Zealand rabbits, SD rats). A large number of scaffolds represented by ion-doped bioceramics, composite polymer scaffolds have obtained stable *in vivo* data on infection clearance, new bone formation and biosafety at 4/8/12 weeks (Zhao et al., 2022; Zhao et al., 2025). This is the main body of current basic research, and no clinical trial has been carried out.
③ Small-scale clinical application (clinical trials):Preliminary clinical exploration with limited sample size (single center, <50 cases). As reported in this review: antibiotic-loaded 3D printed porous titanium alloy scaffolds, PEEK/HA composite scaffolds for maxillofacial defects, and degradable Mg-Zn alloy scaffolds have been applied in single-center small-sample trials. The 12-month follow-up data confirmed good efficacy, but multi-center large-sample verification is still lacking.
④ Approved commercial clinical products:Up to now, no multifunctional 3D printed anti-infective bone scaffolds integrating antibacterial, osteogenic and vascularization functions have obtained medical device registration approval and formal market access. Only traditional 3D printed pure bone repair scaffolds (without independent antibacterial function) have been approved for clinical use in orthopedics and stomatology. All multifunctional anti-infective scaffolds are still in the research and trial stage (Qiao et al., 2022) (Table 3).

**TABLE 3:** Core evaluation indicators and methods of the multi-functional anti-infection bone scaffold.

Evaluation category	Core evaluation indicators	Common detection methods	Qualified judgment criteria for infectious bone defects	Threshold basis & references
Physicochemical Properties	Mechanical strength, porosity, degradation rate, release behavior	Universal testing machine, micro-CT, SEM, ICP-MS	Mechanical strength matches the host bone, porosity 60%–85%, degradation matches bone regeneration, sequential release of antibacterial components	Pore range verified by infectious bone defect model (Zhao et al., 2022); mechanical/degradation standard (Zhao et al., 2025)
*in vitro* Antibacterial Performance	Bacteriostatic rate, biofilm inhibition rate, antibacterial effect on drug-resistant bacteria	Colony counting, bacteriostatic zone, crystal violet staining	Bacteriostatic rate >90% against common clinical pathogenic bacteria, biofilm inhibition rate >80%, effective against MRSA	Universal *in vitro* antibacterial evaluation threshold for orthopedic scaffolds (Yin et al., 2025; Beenken et al., 2021)
*in vitro* Osteogenic Performance	Cell proliferation, ALP activity, mineralization capacity, osteogenic gene expression	CCK-8, ALP/Alizarin Red staining, qPCR	Excellent cytocompatibility in infectious environment, significantly increased ALP activity, upregulated Runx2/OCN expression	Osteogenic evaluation standard for infected microenvironment (Gao et al., 2024)
*in vitro* Angiogenic Performance	Tube formation, cell migration, vascular gene expression	Tube formation assay, Transwell, immunofluorescence	Significant tube formation capacity, obviously upregulated VEGF/CD31 gene expression	Angiogenic detection threshold for bone scaffolds (Qu et al., 2025)
*in vivo* Evaluation	Infection clearance rate, new bone volume, vascular density, inflammatory response	Infectious animal model, micro-CT, histomorphology	Complete infection clearance, abundant new bone and blood vessels, mild inflammatory response, no recurrence	*in vivo* comprehensive evaluation standard for chronic osteomyelitis scaffolds (Qiao et al., 2022)

The above criteria are divided into verified experimental indicators and ideal clinical indicators. The porosity range (60%∼85%), bacteriostatic rate (>90%) and biofilm inhibition rate (>80%) have been fully verified in multiple animal and *in vitro* experiments, which are applicable to polymer, bioceramic and composite scaffolds. The indicators such as “abundant new bone and blood vessels” and “mild inflammatory response” are ideal clinical evaluation standards, which are descriptive indicators for comprehensive judgment in in vivo experiments and clinical follow-up. For metal load-bearing scaffolds, the porosity can be appropriately reduced to 50%–∼70% to ensure mechanical strength, and the above thresholds allow dynamic adjustment according to scaffold material and application site.

## 5 Key challenges and targeted strategies for clinical translation

### 5.1 Core bottlenecks in clinical translation

The complex pathological microenvironment and multi-functional repair requirements of infectious bone defects lead to multiple bottlenecks that restrict the clinical translation and large-scale application of 3D-printed anti-infective bone scaffolds. First, balanced multi-property integration remains difficult. In the infectious microenvironment, it is challenging to synchronize mechanical strength and high porosity, potent antibacterial activity and favorable biocompatibility, as well as degradation rate and bone regeneration speed (Goldmann, 2021). This contradiction is particularly prominent in load-bearing bone defects. Second, uncontrolled release of bioactive components often occurs. Burst release or insufficient sustained release of antibacterial agents, osteogenic and angiogenic factors disrupt the temporal synergy of the tri-functional system. Antibiotic-loaded scaffolds carry the risk of bacterial resistance, while long-term biosafety of inorganic antibacterial ions is still insufficiently verified. Third, standardized evaluation systems are lacking. There are no unified infectious bone defect models or normalized detection indicators, making cross-study comparison difficult. Large-sample, multi-center clinical trials with long-term follow-up are scarce, leading to insufficient evidence on long-term efficacy. Fourth, high costs and technical barriers limit industrialization. High-precision 3D printers and medical-grade biomaterials are expensive; the preparation of multi-functional composite scaffolds involves complex processes and interdisciplinary collaboration, further hindering clinical translation (Patel et al., 2025).

### 5.2 Targeted strategies for promoting clinical translation

1. Manufacturing repeatability and GMP management: Mass production of 3D printed scaffolds must follow Good Manufacturing Practice (GMP) for medical devices. Standardize raw material batch inspection, printing parameter solidification, and post-processing procedures to eliminate performance differences between different batches of personalized implants. For patient-specific customized scaffolds, establish a complete process flow of “CT/MRI data collection → digital modeling → 3D printing → post-treatment → quality inspection → clinical delivery”.
2. Sterilization and quality control: Common sterilization methods for bone scaffolds include ethylene oxide sterilization and gamma ray irradiation. It is necessary to verify that the sterilization process will not damage antibacterial components, active factors and scaffold structure. Establish a full-process quality control system: detect mechanical properties, porosity and drug release performance for each batch of products.
3. Regulatory classification and approval: Multifunctional 3D printed anti-infective bone scaffolds belong to Class III high-risk medical devices in China, requiring strict pre-clinical safety evaluation, multi-center clinical trials and long-term follow-up data before registration and marketing. Intelligent response scaffolds and cell-loaded 3D bioprinting scaffolds face higher regulatory thresholds (Zhuo et al., 2020).
4. Long-term biosafety and degradation monitoring: For degradable scaffolds and ion-doped materials, establish *in vivo* degradation dynamic monitoring technology (micro-CT, imaging tracing) to track scaffold degradation rate, ion metabolism and tissue response for more than 2 years, and evaluate the risk of long-term tissue accumulation and chronic inflammation.
5. Cost-benefit optimization: High-precision 3D printing equipment and medical-grade biomaterials lead to high single-piece cost. Optimize high-throughput printing processes, develop low-cost composite biomaterials, and realize scale production to reduce the overall treatment cost, so as to meet the popularization demand of grassroots medical institutions (Holden et al., 2026).

## 6 Future development trends

Driven by clinical demands for infectious bone defect repair, 3D-printed anti-infective bone scaffolds are evolving toward intelligentization, precise regulation, and clinical translation. Three major development directions can be anticipated (Table 4).

**TABLE 4:** Future development trends and clinical value of 3D printed anti-infection bone supports.

Development trend	Core content	Clinical value in infectious bone defects	Clinical implementation feasibility	Manufacturing & regulatory key points
Smart Responsive Scaffolds	On-demand release triggered by infectious microenvironment, photothermal/photodynamic intelligent antibacterial	Precise targeted anti-infection, reduced drug resistance and toxicity, improved repair efficiency	Medium feasibility; partial systems completed preclinical verification, need stability optimization	Strict control of drug release consistency; comply with class III medical device supervision
4D Printing Technology	Shape memory scaffolds, dynamic structural transformation to adapt to the defect microenvironment	Simplify surgery, improve implant adaptability and tissue integration	Low feasibility; mostly laboratory concept, lack of *in vivo* long-term data	Shape stability verification; high requirement for material batch consistency
Immune-Multifunctional Synergy	Immune regulation, macrophage M2 polarization, inflammatory microenvironment modulation	Optimize the infectious regeneration microenvironment, accelerate bone repair and angiogenesis	Medium feasibility; immune regulation materials mature, combined multifunction needs optimization	Cell factor activity detection; biosafety evaluation of immunomodulatory components
Standardization and Industrialization	Unified evaluation standards, low-cost high-throughput printing, medical device certification	Promote large-scale clinical application, reduce treatment cost	High feasibility; existing production lines can be upgraded	GMP production standard, unified quality control system, batch repeatability testing

This table sorts four promising research directions of 3D printed anti-infective bone scaffolds. Clinical implementation feasibility is classified by preclinical verification progress, and key manufacturing and regulatory requirements correspond to the supervision standards of Class III medical devices for orthopedic implants in China.

First, intelligent responsive systems and 4D printing will be deeply integrated. Infection microenvironment-triggered drug delivery and photothermal/photodynamic antibacterial designs enable on-demand release of antibacterial agents, reducing drug resistance and cytotoxicity. 4D printing with shape-memory materials allows dynamic structural transformation to better adapt to complex defect geometries, thereby improving implant fitting and tissue integration (Choudhury et al., 2024). For instance, the pH-responsive PLGA/antibiotic microcarrier system and bacteria-specific enzyme cleavage response scaffold have achieved stable on-demand drug release in chronic osteomyelitis models, which are the most potential intelligent technologies for subsequent clinical transformation.

Second, immune regulation will be combined with tri-functional synergy. By introducing immunomodulatory functions to promote macrophage polarization toward the M2 phenotype, the inflammatory microenvironment can be remodeled favorably. This forms a multi-level cooperative network of immune regulation–antibacterial–osteogenic–angiogenic activities, further enhancing repair efficiency in infectious sites (Shiratori et al., 2017).

Third, clinical standardization and industrialization will be accelerated. The establishment of unified standards for materials, fabrication, evaluation, and clinical trials, together with low-cost high-throughput printing and medical device certification, will lower barriers to clinical use and promote large-scale clinical application of 3D-printed scaffolds (Lin et al., 2023).

## 7 Conclusion

As one of the most intractable clinical challenges in orthopedics, the repair of infectious bone defects fundamentally requires the simultaneous achievement of infection elimination, bone regeneration, and vascular reconstruction in a complex infectious microenvironment. The spatiotemporal synergy of antibacterial, osteogenic, and angiogenic functions represents the key to breaking through current therapeutic limitations and realizing integrated repair. With its unique advantages of personalized customization, precise structural control, and wide material compatibility, 3D printing has become an ideal platform for constructing multifunctional anti-infective bone scaffolds, providing a novel technical strategy for addressing this long-standing clinical dilemma.

Different from existing reviews that merely focus on single function, single material, or single fabrication technology, this review takes the spatiotemporal synergy of antibacterial–osteogenic–angiogenic tri-functions as the core theme for the first time. Based on the authentic pathological characteristics of infectious bone defects, we systematically summarize the material systems, anti-infective strategies, 3D printing techniques, biomimetic structural designs, and standardized evaluation systems of 3D-printed anti-infective bone scaffolds. Throughout the review, we emphasize the particularity of the infectious microenvironment and substantially strengthen the discussion of clinical translation by supplementing clinical evidence, translational bottlenecks, and targeted regulatory strategies, thus forming a complete framework covering basic research, mechanism interpretation, structural design, and clinical translation.

At present, remarkable progress has been made in the fundamental research of 3D-printed anti-infective bone scaffolds, with continuous breakthroughs in material hybridization, functional modification, and bionic structure construction, laying a solid experimental and theoretical foundation for the treatment of infectious bone defects. Nevertheless, several critical obstacles remain before large-scale clinical application: the difficulty in balancing multifunctional properties, the lack of precise spatiotemporal control over bioactive component release, the absence of unified standardized evaluation systems, and the high costs and technical barriers associated with clinical translation and industrialization. These issues will remain the core directions for future investigation.

Looking forward, with the rapid advancement of smart responsive materials, 4D printing, immunomodulatory technology, and interdisciplinary integration, 3D-printed anti-infective bone scaffolds will gradually evolve toward intelligence, precision, standardization, and industrialization. By developing microenvironment-responsive controlled-release systems, establishing immune–osteogenic–angiogenic multi-level synergy, constructing unified clinical evaluation criteria, and promoting low-cost industrial manufacturing, such novel scaffolds are expected to be widely applied in clinical practice. They will provide a safe, efficient, personalized, and integrated therapeutic paradigm for infectious bone defects, significantly improve the success rate of bone defect repair, and enhance the long-term prognosis and quality of life of patients, demonstrating important scientific significance and broad clinical application prospects.

In addition to material and technological innovation, the industrialization and clinical popularization of such scaffolds also rely on the improvement of medical device regulatory systems, the implementation of GMP production specifications, the standardization of sterilization and quality control procedures, and the reduction of comprehensive manufacturing costs. Only by building a complete chain of “basic research - standardized production - regulatory approval - clinical application” can the translational value of 3D printed multifunctional bone scaffolds be fully realized.
